# RETIREMENT TRAJECTORIES AND HEALTH IN JAPAN

**DOI:** 10.1093/geroni/igad104.3008

**Published:** 2023-12-21

**Authors:** Masaaki Mizuochi, James Raymo

**Affiliations:** Nanzan University, Nagoya, Aichi, Japan; Princeton University, Princeton, New Jersey, United States

## Abstract

The relationship between retirement and health is a key issue in aging societies. However, it is unclear whether the nature of retirement from full-time regular employment makes a difference in subsequent health. This study examines the relationship between the trajectory of retirement from full-time regular employment and subsequent health. Using the Longitudinal Survey of Middle-aged and Elderly Persons 2005–2019 collected in Japan, we employ sequence analysis to identify distinct retirement trajectories from full-time regular employment between ages 59 and 66. We then estimate the causal relationship between retirement trajectories and health using instrumental variable methods. Sequence analysis classifies the retirement trajectories into four groups: gradual retirement, abrupt retirement, continued non-standard employment, and continued regular employment. Instrumental variable estimation shows no statistically significant differences in the effects of these retirement trajectories on health, but suggests that gradual retirement may be more beneficial to health than other three retirement trajectories. Several robustness checks also show no statistical difference in the effect of retirement trajectories on health. These findings provide an empirical foundation for understanding the possible implications of heterogenous retirement pathways for health in later life and labor market policies in very old populations.

